# The emerging role of exosomes in communication between the periphery and the central nervous system

**DOI:** 10.1002/mco2.410

**Published:** 2023-10-30

**Authors:** Wenxiu Han, Hailiang Zhang, Lei Feng, Ruili Dang, Jing Wang, Changmeng Cui, Pei Jiang

**Affiliations:** ^1^ Translational Pharmaceutical Laboratory Jining First People's Hospital Shandong First Medical University Jining P. R. China; ^2^ Institute of Translational Pharmacy Jining Medical Research Academy Jining P. R. China; ^3^ Department of Neurosurgery Jining First People's Hospital Shandong First Medical University Jining P. R. China; ^4^ Department of Neurosurgery Affiliated Hospital of Jining Medical University Jining P. R. China

**Keywords:** Blood—brain barrier (BBB), central nervous system (CNS), communication, exosome, periphery

## Abstract

Exosomes, membrane‐enclosed vesicles, are secreted by all types of cells. Exosomes can transport various molecules, including proteins, lipids, functional mRNAs, and microRNAs, and can be circulated to various recipient cells, leading to the production of local paracrine or distal systemic effects. Numerous studies have proved that exosomes can pass through the blood—brain barrier, thus, enabling the transfer of peripheral substances into the central nervous system (CNS). Consequently, exosomes may be a vital factor in the exchange of information between the periphery and CNS. This review will discuss the structure, biogenesis, and functional characterization of exosomes and summarize the role of peripheral exosomes deriving from tissues like the lung, gut, skeletal muscle, and various stem cell types in communicating with the CNS and influencing the brain's function. Then, we further discuss the potential therapeutic effects of exosomes in brain diseases and the clinical opportunities and challenges. Gaining a clearer insight into the communication between the CNS and the external areas of the body will help us to ascertain the role of the peripheral elements in the maintenance of brain health and illness and will facilitate the design of minimally invasive techniques for diagnosing and treating brain diseases.

## Graphical abstract

1. Exosomes can pass through the blood—brain barrier (BBB) and act as a vital factor in the exchange of information between the periphery and central nervous system (CNS).

2. This review summarizes the role of peripheral exosomes deriving from tissues like the lung, gut, skeletal muscle, and various stem cell types, in communicating with the CNS and influencing the brain's function.

3. Exosomes have the potential to be used for therapeutic purposes, and new strategies for taking advantage of them are being developed.

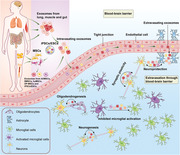

## 1 INTRODUCTION

The central nervous system (CNS), which consists of the brain and spinal cord, is responsible for the control and regulation of the physiological and psychological activities of every other system in the body. Recent studies have demonstrated that the peripheral and central immune systems, organs, such as the heart, lung, and kidney, and the brain, can all interact with each other in both healthy and pathological conditions, thus, making the communication between the periphery and the brain a topic of growing interest.^1^, ^2^, ^3^, ^4^, ^5^ Evidence has demonstrated that brain disorders can cause issues in peripheral organs^6^, ^7^, ^8^ and vice versa; peripheral pathologies can exacerbate a variety of CNS conditions, including Parkinson's Disease (PD), Alzheimer's Disease (AD), hemorrhagic stroke (HS), ischemic stroke (IS), and traumatic brain injury (TBI).^5^, ^9^, ^10^ During the process of interaction between the periphery and CNS, the communicators must play critical roles. Traditionally, the interaction between the periphery and CNS can be facilitated by the autonomic nervous system and neuroendocrine system. The hypothalamus, a high center of these systems, is responsible for regulating basic physiological functions such as digestion and body temperature. Furthermore, peripheral signals, such as gastrointestinal hormones, insulin, and leptin, are transported to the brain to regulate innate behaviors such as feeding, as well as emotional and cognitive functions. Additionally, the brain can recognize peripheral inflammatory cytokines, leading to the development of a transient syndrome known as sick behavior, which is characterized by fatigue, reduced physical and social activity, and cognitive impairment.^11^, ^12^

In recent years, exosomes have drawn much attention due to their potential to facilitate intercellular communication and mediate between the periphery and CNS. Exosomes, which can be found in different bodily secretions, like plasma, urine, saliva, synovial fluid, amniotic fluid, and pleural effusions, comprise several functional elements including lipids, proteins, mRNA, and microRNA.^13^, ^14^ Exosomes, secreted from cells, traverse the circulation and are taken up by recipient cells, acting as intercellular communicators and mediators of information transmission between cells.^15^, ^16^ With their lipophilic properties, exosomes can cross the blood—brain barrier (BBB), affecting brain function.^17^, ^18^ Exosomes originating from the periphery can be transported to the brain, and exosomes from the brain can also be located in the periphery.^19^ Growing evidence suggests that exosomes have significant roles in neurological diseases, particularly in neuroinflammation and neurodegeneration,^20^, ^21^ which are closely associated with the control of angiogenesis and neurogenesis in various neurological diseases.^22^, ^23^ Studies have demonstrated that exosomes from the peripheral blood of aged mice can affect gene expression in the brains of younger animals, with a robust response of astrocyte cells indicated by an increased expression of the marker, Gfap.^24^ Exosomes originating from the body's peripheral regions may enable the actions of cytokines, insulin, and irisin to manifest in the CNS during healthy and pathological circumstances.^16^, ^25^

Exosomes are seen as an integral factor in transmitting messages between the CNS and peripheral areas. Exosomes contain various lipids and proteins, the quantity of which is contingent on the cell type and conditions. Additionally, exosome proteins are distinctive to the cell type, thus, determining their purpose and function. This review will discuss the structure, biogenesis, and functional characterization of exosomes, and highlight the role of peripheral exosomes deriving from tissues like the lung, gut, skeletal muscle, and various stem cell types in the CNS, and focus on how this affects the brain's functioning. By deepening our understanding of the interplay between the periphery and the CNS, we can better understand the impact of external factors on the brain's health and diseases and, consequently, create more effective, minimally invasive approaches for the diagnosis and treatment of brain diseases.

## 2 EXOSOME STRUCTURE, BIOGENESIS, AND FUNCTIONAL CHARACTERIZATION

### 2.1 Exosome definition and nomenclature

It has been 50 years since Wolf first identified extracellular vesicles (EVs) in plasma, which he referred to as “platelet dust.”^26^ Subsequent studies have indicated that all biological fluids and cell lines grown in vitro secrete vesicles, with the amount varying between them.^27^, ^28^, ^29^ Three distinct types of EVs have been identified, characterized by their release mechanism and size: exosomes, microvesicles (MVs), and apoptotic bodies. Exosomes, the smallest type of EVs, which are nanoscale vesicles with a bilayer structure, are released from cells through a multistep exocytotic process.^15^, ^30^, ^31^ These particles range in size from 30 to 150 nm. Ectosomes, also known as MVs, are EVs with a size ranging from 100 to 1 mm that are generated by the plasma membrane's direct outward budding. Apoptotic bodies, EVs with diameters of 50 to 5000 nm, are released by cells going through apoptosis.^15^, ^30^, ^32^ As nanovesicles of similar size to exosomes and released by budding off the plasma membrane are also denoted as exosomes,^33^, ^34^ there is no agreement on the nomenclature of exosomes and MVs. In this review, we have used the terms “exosome” and “ectosome/microvesicle” for particles with biogenesis that have been proven in the referenced publication and the more general term “EVs” when the distinction is not clear.

### 2.2 Exosome composition and biogenesis

**FIGURE 1:**
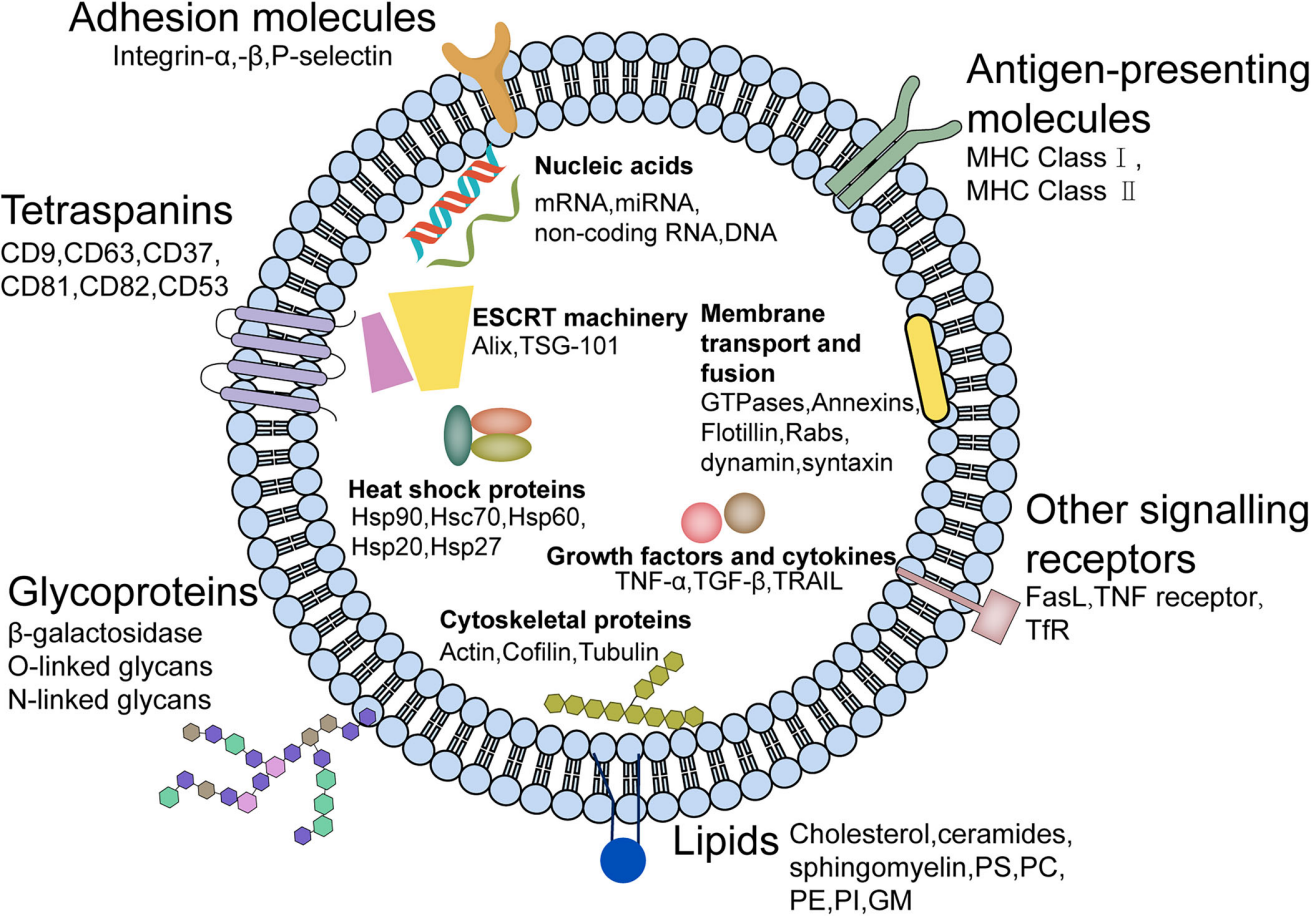
The composition of exosomes. Exosomes contain transmembrane proteins like tetraspanins (CD9, CD63, CD37, CD81, CD82, CD53), adhesion molecules (Integrin‐α,‐β, P‐selectin), antigen‐presenting molecules (MHC Class I, MHC Class II), glycoproteins (β‐galactosidase, O‐linked glycans, N‐linked glycans), and other signaling receptors (FasL, TNF receptor, TfR); as well as lumen proteins such as cytoskeletal proteins (actin, cofilin, tubulin), heat shock proteins (Hsp90, Hsc70, Hsp60, Hsp20, Hsp27), membrane transport and fusion proteins (GTPases, annexins, flotillin, Rab GTPases, dynamin, syntaxin), ESCRT machinery (alix, TSG‐101), and growth factors and cytokines (TNF‐α, TGF‐β, TRAIL); lipids (cholesterol, ceramides, sphingomyelin, PS, PC, PE, PI, GM); and nucleic acids (mRNA, miRNA, noncoding RNA, DNA). FasL, Fas ligand; TNF, tumor necrosis factor; TfR, transferrin receptor; Hsp, heat shock protein; TSG, tumor susceptibility gene; TGF, transforming growth factor; TRAIL, TNF‐related apoptosis inducing ligand; PS, phosphatidylserine; PC, phosphatidylcholine; PE, phosphatidylethanolamine; PI, phosphatidylinositol; GM, gangliosides.

Generally, the composition of exosomes (Figure 1) is contingent on their origin and cell types. Multiomics studies have demonstrated that exosomes contain a range of biomolecules including proteins, lipids, and nucleic acids.^35^, ^36^, ^37^ Studies have established that exosomes are composed of 4563 proteins, 764 miRNAs, 1639 mRNAs, and 194 lipids.^38^ Notably, the most recurrent membrane‐bound and cytosolic proteins identified in exosomes include members of the tetraspanin family (CD9, CD63, CD37, CD81, CD82, and CD53), endosomal sorting complex required for transport (ESCRT) proteins (apoptosis‐linked gene 2‐interacting protein X [Alix], tumor susceptibility gene 101 [TSG101]), integrins, heat shock proteins (Hsp), actin, and flotillins.^39^, ^40^ Exosomes contain proteins, including Rab11, Rab27a, Rab27b, GTPases, ADP ribosylation factors, and annexins, which may facilitate the fusion of MVB with the cell membrane and the exosome secretion process.^41^ Besides the proteins that are commonly found, exosomes may also contain proteins that are specific to the cell type and also vary depending on the physiological state.^42^ Exosomes are highly enriched in cholesterol, sphingomyelin, glycosphingolipid, phosphatidylserine, and ceramide, which are all essential for the maintenance of exosome morphology, biogenesis, and homeostasis in the recipient cells.^43^ Recently, exosomes have been identified to contain nucleic acids such as DNA, miRNA, and noncoding RNA.^44^ Through the exosomal transfer of RNA molecules from parent cells to recipient cells, an alternative form of intercellular communication has been studied in great detail, particularly with regard to the communication between the CNS and the periphery.

**FIGURE 2:**
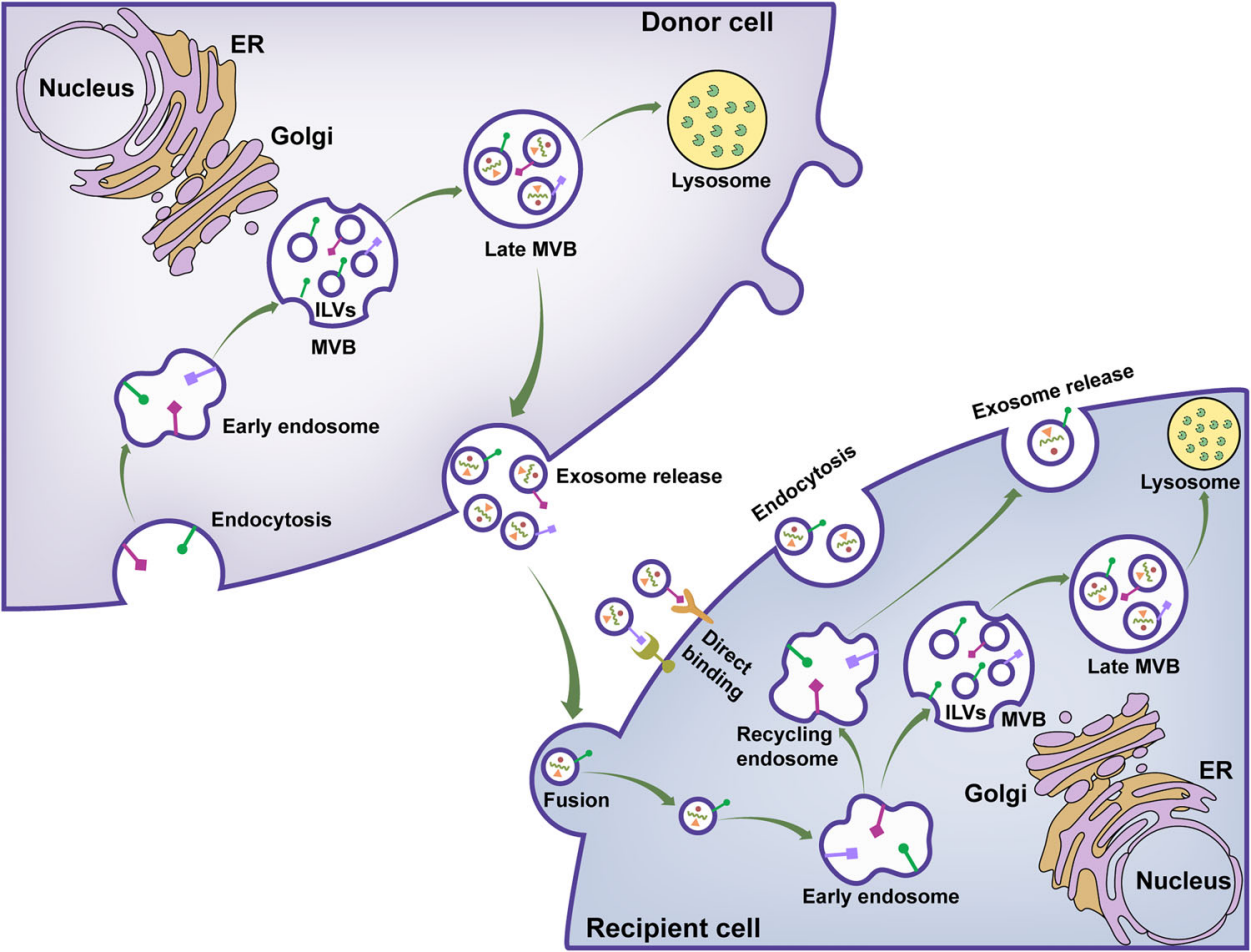
Exosome biology. Exosomes originate from the late endocytic pathway through the formation of intraluminal vesicles (ILVs) and are then released from the cell surface by exocytosis. Upon targeting the recipient cell, exosomes engage with the surface molecules of the recipient cell to initiate downstream signaling or merge with the plasma membrane to unload their contents. Additionally, the internalization of exosomes is achievable through multiple routes, leading them to the early endosome, late endosome, or MVB. The exosome contents may be released into the nucleus or ER, traverse the cytosol, or be broken down in the lysosomes. Moreover, the recycling endosome allows exosomes to be rereleased into the external space. MVB, multivesicular body; ER, endoplasmic reticulum.

The biogenesis of exosomes involves the inward folding of early endosomes, thereby creating multivesicular bodies (MVBs) that contain intraluminal vesicles. As documented by Johnstone et al. (1987),^45^ the merger of MVBs with the plasma membrane results in the dispersal of exosomes into the nearby environment. All cell types are able to produce exosomes, which are formed within the cell through the inward budding of endosomes, producing MVBs (Figure 2). By fusing the MVBs with the plasma membrane, exosomes can be released into the external environment.^46^, ^47^, ^48^ The plasma membrane undergoes invagination, forming a cup‐shaped structure enclosing the cell‐surface and soluble proteins connected to the external environment. This leads to the formation of an early‐sorting endosome (ESE), which can be combined with an existing ESE in some cases. The trans‐Golgi network and endoplasmic reticulum collaborate in the formation and composition of the ESE, which then progress to late‐sorting endosomes and eventually become MVBs. The endosomal limiting membrane folds inward, forming MVBs which contain several intraluminal vesicles (ILVs). The MVBs can either fuse with the plasma membrane and release the ILVs as exosomes or fuse with lysosomes or autophagosomes for degradation.^15^, ^49^, ^50^ Several proteins, including Ras‐related GTPase Rab, Sytenin‐1, TSG101, Alix, ESCRT proteins, phospholipids, tetraspanins, ceramides, sphingomyelinases, and soluble NSF attachment protein receptors (SNARE) complex proteins, have been identified to be involved in the process of exosome biogenesis. However, the exact role and rate‐limiting functions of these proteins in the origin and biogenesis of exosomes must be further explored, particularly in vivo.^51^, ^52^, ^53^

### 2.3 Recipient cell uptake of exosomes

**FIGURE 3:**
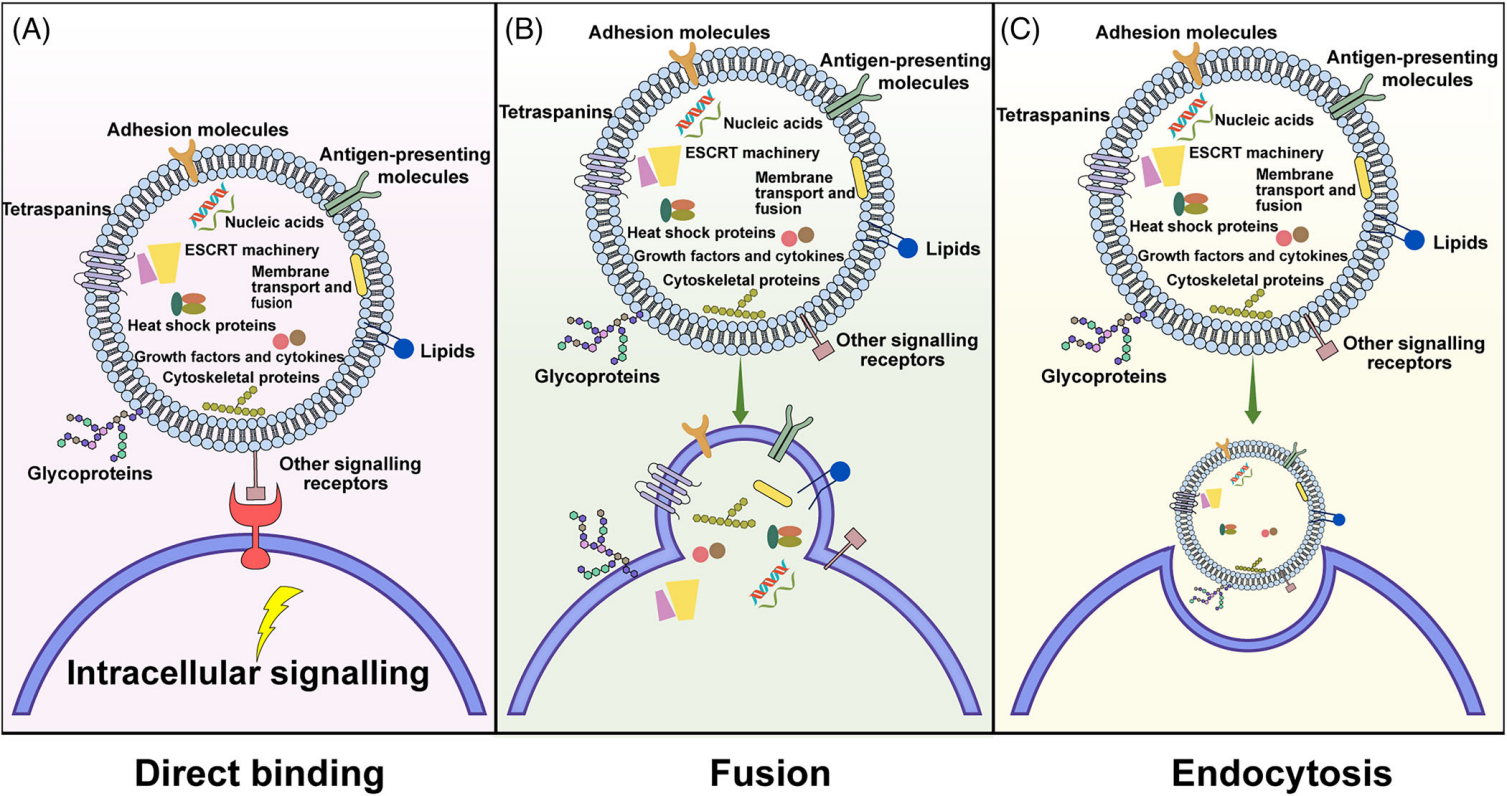
Exosomal signaling through direct binding, membrane fusion, or endocytosis. (A) On reaching the target cells, molecules situated on the exosome's surface function as ligands, connecting to the receptors on the plasma membrane of the target cells, thus initiating an intracellular signaling cascade inside the recipient cell. (B) The exosome membrane fuses with the plasma membrane, thereby allowing the exosome contents to be released into the cytosol immediately. (C) The plasma membrane's deformational motion enables the transport of exosomes into the recipient cell, thereby activating signal transduction.

As shown in Figure 3, upon reaching the recipient cell, exosomes can activate signaling through direct contact with extracellular receptors, be absorbed by merging with the plasma membrane, or be taken up internally.

#### 2.3.1 Direct interaction

Dendritic cells release exosomes, which can interact with the receptors on the recipient cells and trigger signaling pathways that control immunomodulatory and apoptotic activities. Studies have revealed that exosomes can induce T‐lymphocyte activation via the Major Histocompatibility Complex (MHC)‐peptide complex,^54^ and they have the capacity to bind Toll‐like receptor ligands on bacterial surfaces to activate bystander dendritic cells, thereby intensifying immune responses.^55^ Studies have demonstrated that exosomes derived from umbilical cord blood, containing tumor antigens, such as MHC‐I, MHC‐II, and tetraspanins (CD34, CD80), have the capacity to trigger T‐cell proliferation and, consequently, show antitumor capabilities.^56^ Additionally, dendritic cells can induce apoptosis in tumor cells by releasing exosomes, which bind to tumor necrosis factor (TNF) receptors and stimulate caspase activation. This is achieved through the expression of ligands such as TNF, Fas ligand (FasL), and TNF‐related apoptosis inducing ligand (TRAIL) on the surface of the cells.^57^

#### 2.3.2 Fusion with the plasma membrane

Exosomes have the capability to fuse with the plasma membrane and discharge their contents directly into the interior of the target cells. The process is initiated by the formation of a hemifusion stalk between the hydrophobic lipid bilayers of the exosome and plasma membrane, and then proceeds to expand, forming a unified structure. Studies on cell membrane fusion^58^ have demonstrated that SNAREs and Rab protein families are likely to be responsible for this fusion.^59^, ^60^ The exosome surface is composed of lipid raft‐like domains, integrins, and adhesion molecules, facilitating interaction, attachment, and the combining of membranes with the target cell.^61^, ^62^, ^63^ By utilizing exosomes that incorporate the lipophilic dye octadecyl rhodamine B (R18), it is possible to distinguish between endocytosis and fusion since the fluorescent signal is weakened when it combines with recipient membranes that have not been labelled.^64^ Studies have noted this occurrence in dendritic and tumoral cells.^65^, ^66^ Speculation exists that the acidic nature of tumor microenvironments, which results in increased rigidity and sphingomyelin levels, could promote exosome fusion, thus, making it a plausible pathway for tumor cells.^67^

#### 2.3.3 Endocytosis

Recent studies have suggested that endocytosis is the primary mechanism for the uptake of exosomes. Depending on the cell type, the internalization of exosomes by recipient cells can be achieved by clathrin‐, caveolin‐, and lipid raft‐mediated endocytosis.^68^ Upon endocytosis, exosomes may either fuse with endosomes or be transported to lysosomes for degradation.^62^ Generally, exosomes are taken up by the recipient cell, followed by the release of their cargo.^69^, ^70^, ^71^ This process is rapid, temperature‐sensitive, and inhibited by low temperatures.^72^ The endocytic pathways are also involved in the internalization of exosomes.

### 2.4 Exosome isolation, identification, and storage

In the past few decades, the exosome isolation technique has also undergone rapid development. At present, exosomes are usually isolated by a combination of methods, such as ultracentrifugation, ultrafiltration (UF), size‐exclusion chromatography (SEC), polymer‐based precipitation, immunoaffinity capture, and microfluidic separation, based on their size, density, and immunoaffinity. Ultracentrifugation, which is simple, low‐cost, and has a high purity rate, is typically the first step and is considered the gold standard.^73^ However, this process has some drawbacks, such as the need for a large volume of biological fluid, long processing times, and limited reproducibility, as well as the potential for the exosomes to be damaged by excessive force. UF can separate exosomes of specific sizes, and is simple to operate, and is suitable for the analysis of large quantities of biological samples. However, membrane blockage is its evident limitation, which can shorten the life of the expensive membrane and reduce isolation efficiency, thereby leading to the misinterpretation of test results.^74^ Tangential flow filtration (TFF) technology is an emerging exosome isolation technique with the ability to solve this problem effectively.^75^, ^76^, ^77^ Although few limitations still exist in current TFF technology, we believe that the application of TFF technology in the field of exosome isolation will increase significantly, with the continuous advancement of fluid mechanics and materials science. SEC shows a significant advantage of preserving the integrity and natural biological activity of the isolated exosomes.^78^ In addition, SEC is more user friendly, less time consuming, better repeatability, and has greater potential for high‐throughput industrial applications than other isolation methods,^79^, ^80^ which makes it easier to adapt to most laboratories. We also expect the combination of UF and SEC can facilitate the preparation of clinical‐grade exosome products in the future.

Compared with ultracentrifugation or SEC, polymer precipitation method exhibits the advantages of simplicity, scalability, high yield, and no deformation of exosomes,^81^ which has potential advantages for future clinical research. However, it is time consuming and the purity of obtained exosomes is lower,^82^, ^83^ which can affect downstream analysis. Hence, it is not considered as an appropriate method for the descriptive or functional analysis of exosomes. The immunoaffinity capture method separates exosomes based on immunoaffinity techniques, the exosomes isolated by this method exhibit higher specificity and yield.^84^, ^85^ Although the use of antibodies can shorten the isolation time, improve the exosome purity, and enable the acquisition of specific exosome components.^86^ However, due to the specificity and high price of antibodies, this method is only suitable for small‐sample studies, and the exosomes isolated can usually be used for diagnostic purposes, but are not conducive to exosome‐based functional research and various therapeutic applications.^87^, ^88^ The microfluidic technique is a high‐throughput method because it consumes less sample volume, reagents, and isolation time.^89^ However, the relatively low efficiency of microfluidic techniques can adversely affect downstream assessments and lead to inaccurate test results. It is worth noting that a simple, fast, and label‐free chitosan‐modified shuttle flow microchip has been developed to capture and release exosomes from human serum flexibly within 15 min with a high purity of over 90% and a high recovery ratio of over 84%, which is impossible for ultracentrifugation methods.^90^ Therefore, it is expected that improvements in microfluidic devices will certainly bring more opportunities to the medical field in the future, and simple, continuous, and rapid exosome isolation can be achieved via this method. Despite the effectiveness of all the methods above, a combination of multiple separation techniques may be required to isolate the desired exosomes due to the presence of various subtypes.

Exosomes are small, low‐density, and heterogeneous particles, making it difficult to isolate them using a single method.^30^ Multiple identification techniques are required to accurately assess their physical and biological properties, size distribution, concentration, and integrity. Commonly used techniques include transmission electron microscopy (TEM), western blotting, confocal microscopy, and nanoparticle tracking analysis (NTA).^91^ Utilizing TEM, it is possible to identify the surface characteristics of exosomes, such as size and form, differentiate single exosomes from other particles of similar size, and measure their quality and concentration.^92^ Western blotting is a technique used to measure the amount of target proteins in a sample, with the benefit of requiring only a small sample size and being highly efficient.^93^ Characteristic transmembrane proteins, such as CD9, CD63, and CD81, can be accurately identified through western blotting.^94^ Confocal imaging technology enables the acquisition of 3D images of exosomes, thus allowing for clear visualization, qualitative and quantitative assessment, and the capacity to differentiate between particular fluorescent signals and artificial signals from exosomes.^95^ NTA is a convenient tool for semiquantitative particle characterization, allowing for the analysis of exosome size and quantity.^96^ This method involves light scattering measurements and tracking the Brownian motion of irradiated single particles using an optical microscope equipped with a high‐definition camera.^97^

Cryopreservation (4°C for the short term and −80°C for the long term) is the most commonly used method for storing isolated exosomes.^98^ However, it is important to avoid repeated freeze‐thaw cycles to maintain their integrity. The half‐life of exosomes in circulation can vary depending on various elements such as the method of administration, size, and the existence of proteins or enzymes that could break them down. The half‐life of intravenous exosomes is limited, with a duration of several minutes to a few hours, thus, limiting their use in certain applications.^99^ To enhance the longevity and stability of exosomes, multiple approaches have been investigated including coating them with polymers, embedding them in hydrogels, and loading them with protective substances. Through these approaches, degradation can be reduced, circulation time can be increased, and therapeutic potential can be improved.^100^

## 3 EXOSOMAL TRANSPORT ACROSS THE BBB

The BBB is composed of neurovascular units, serving as a connection between the CNS and the exterior surroundings. Its specialized tight and adherent junctions of brain endothelial cells provide physical protection and act as a transport interface, a secretory body, and a metabolic barrier, all of which are necessary for selectively blocking the entrance of substances from the blood to the CNS, and sustaining the brain homeostasis.^101^

For peripheral and CNS communication to be successful, it is necessary to be able to penetrate the BBB. Findings have indicated that exosomes possess lipophilic qualities and can reciprocate between the brain and the circulatory system, thus, allowing the delivery of peripheral substances into the CNS.^17^, ^18^, ^102^ Furthermore, exosomes located in the periphery can also interact with the BBB, thus, modifying its features to facilitate the crossing process.^103^ Exosomes originating from leukemia could be of great value, as they can modify the integrity of the connection between endothelial cells in the body and even facilitate the passage into the brain.^104^ It has been observed that fluorescently labeled exosomes, when injected peripherally, can be detected in the brains of recipient mice in vivo.^105^ The infusion of exosomes derived from aged mice into young animals leads to the activation of glial cells and extreme astrogliosis.^24^ Preclinical models have been used to assess the efficacy of exosomes in delivering small RNAs and proteins across the BBB. Exosomes containing a short interfering siRNA for glyceraldehyde 3‐phosphate dehydrogenase (GAPDH) have been demonstrated to be able to cross the BBB and effectively reduce the expression of GAPDH in microglia, neurons, and oligodendrocytes.^106^ Exosomes carrying siRNA for α‐synuclein, which are administered systemically, have been demonstrated to be clinically relevant as they effectively reduce α‐synuclein expression in the brains of mice,^107^ thus, making exosomes clinically relevant. It has been established through all this research that exosomes have the ability to traverse the BBB.

Mechanistically, despite the fact that it has yet to be fully comprehended, it is speculated that exosomes are able to traverse the BBB, facilitated by their small size and lipid membranes.^108^, ^109^ And, endothelial cells are thought to have the ability to take up and transfer exosomes through receptor‐mediated or adsorptive transcytotic mechanisms due to not only the presence of transmembrane ligands on exosomes but also the presence of specialized BBB transporters and receptors.^110^ As described before, upon reaching the target endothelial cell, the particles can initiate signaling by connecting to cell surface receptors. Alternatively, they can be absorbed by merging with the plasma membrane, or they can be taken up internally. Meanwhile, studies have demonstrated that exosomes have an effect on the integrity and permeability of BBB, thus, facilitating the cross‐linking of BBB for exosomes. For example, studies show breast cancer cell‐derived exosomes could specifically express miR‐105, which could target the TJ protein ZO‐1 to reduce the integrity of the BBB.^111^ Besides, another study demonstrated for the first time that human dental pulp stem cell‐derived exosomes could increase vascular permeability in the initial stage of acute inflammation in mice model.^112^ In addition, the engineering of exosomes is a promising strategy for the crossing of the BBB. Surface modification and loading of functional groups that target the brain can facilitate the entry of exosomes into the brain. As an example, Alvarez‐Erviti and colleagues demonstrated that small peptides derived from the rabies virus glycoprotein (RVG) could be used to modify exosomes and make them suitable for brain delivery due to their safety and efficiency in crossing the BBB.^113^ Exosomes can, therefore, be seen as potential cellular mediators in the communication between the periphery and CNS.

## 4 PERIPHERAL EXOSOMES COMMUNICATE WITH THE CNS

As stated in the aforementioned description, exosomes are becoming increasingly recognized as a form of intercellular communication and mediation. Researches have showed that neural stem/progenitor cells (NPCs) and induced NPCs (iNPC)‐derived EVs labeled with a lipophilic membrane dye Dil can cross the BBB, and the Dil signals were detected in mouse brains 5 min after the intravenous administration by in vivo fluorescence analyses. Moreover, the brain distribution of NPC and iNPC‐EVs has been analyzed based on the co‐localization between Dil signals and the immunoreactivities of GFAP, Iba1, and Tuj1, implying the uptake of NPC and iNPC‐EVs by neurons, astrocytes, and microglia in the cortex and hippocampus.^114^, ^115^ Similarly, after the infusion of PKH67 dye‐labeled small EVs (sEVs) derived from aged mice into young animals via tail vein, the distribution of sEVs in brain tissue was not homogenous and mainly concentrated in the cortex and the hippocampus, as observed by the agglomeration of green signals in cluster‐like complexes.^24^ Intravenously injected RVG‐targeted exosomes delivered GAPDH siRNA specifically to neurons, microglia, and oligodendrocytes in the brain, resulting in a specific gene knockdown.^106^ All these preclinical models have been used to assess the efficacy and therapeutic potential of exosomes taken up by brain cells including neurons and glial cells.^105^, ^106^, ^116^ Thus, it can be seen that exosomes may be essential in the transmission of information from the periphery to the CNS by means of their capacity to traverse the BBB. In this part, we will highlight the role of exosomes deriving from tissues like the lung, gut, skeletal muscle, and various stem cell types in the CNS, and focus on how this affects the brain's functioning.

### 4.1 Peripheral tissues derived exosomes

#### 4.1.1 Gut

“All disease begins in the gut.”

–Hippocrates of Kos (Hippokrátes ho Kṓos:c. 460‐c. 370 BCE)

The gut—brain axis is a two‐way interaction between the gut and CNS, promoted by the production of neuroactive molecules from the gut microbiota. These molecules, including acetylcholine, GABA, and serotonin, can influence the brain either directly or indirectly.^117^, ^118^, ^119^ It is remarkable that 90% of the serotonin, which is necessary for mood, behavior, sleep, and other functions in the CNS and GI tract, is produced in the gut.^120^ As the link between the gastrointestinal system and the brain is increasingly explored in the research of psychiatric, neurodevelopmental, age‐related, and neurodegenerative disorders, investigations into this connection are becoming more frequent.^121^, ^122^ Exosomes, which are secreted from the gut and gut microbiota, may be essential mediators of the connection between the gut and the brain.^122^, ^123^ Bacterial membrane vesicles are currently regarded as microbiota‐derived EVs since they share characteristic similarities in size, structure, and biological function with EVs derived from mammalian cells.^124^ More recent research revealed that outer‐membrane vesicles (OMVs) derived from the microbiota may cross the BBB and contribute to neuroinflammation and cognitive impairment, which are linked to CNS disorders. The mechanism may involve the transfer of small RNA noncoding RNA elements contained within OMVs into host cells, thereby regulating host gene expression.^125^, ^126^, ^127^, ^128^, ^129^, ^130^ Research has shown that exosomes originating from microbiota can be released into the systemic circulation and pass through the BBB.^131^, ^132^ In vivo imaging shows that *Helicobacter pylori‐* and *Staphylococcus aureus*‐released exosomes can be found in the brain when administered orally or intramuscularly.^133^, ^134^ Immunoblot analysis has demonstrated glial cells and major cerebral vessels of individuals with AD have been observed to contain LPS, a key virulence factor of *Porphyromonas gingivalis* outer membrane vesicles.^135^ Research has proposed that exosomes from the microbiota may be taken up by mesenteric veins, delivered by the hepatic portal vein and liver, and eventually reach the brain through the circulatory system.^136^ These findings imply that microbiota‐derived exosomes may directly affect the CNS and serve as a significant central modulator of the gut—brain—microbiota axis.

It is well known that diabetes mellitus, metabolic syndrome, and obesity are risk factors for the emergence of CNS disorders. Obesity‐induced chronic inflammation, transmitted through the gut—brain axis, is a hallmark of obesity. Furthermore, research has demonstrated that miRNA derived from gut microbiota exosomes is an essential component in the pathogenesis of metabolic diseases and the harm it causes to target organs.^137^, ^138^, ^139^ The activation of the brain‐gut connection by food is beneficial for the brain and metabolism, which is regulated by functional exosomes. Ingestion of garlic exosome‐like nanoparticles has been demonstrated to be effective in reversing the obesity and insulin resistance induced by a high‐fat diet in mice while also enhancing their memory function, glucose tolerance, and insulin sensitivity. The underlying mechanism is associated with inhibiting the microglial c‐Myc‐mediated cGAS/STING/IDO1/AHR inflammatory signaling.^140^ A double‐blind, randomized controlled trial demonstrated that carnosine, found in high amounts in chicken breast meat, can enhance brain function. Research has shown that exosomes derived from Caco‐2 cells treated with carnosine trigger neurite growth in SH‐SY5Y cells, thus, establishing the connection between the gut and the brain.^141^ The growing evidence of exosomes’ role in the gut—brain axis has revolutionized our recognition of systemic influences on brain function and may open opportunities for novel therapeutic approaches to mood issues, neurodevelopmental and cognitive disorders.

#### 4.1.2 Skeletal muscle

Studies have shown that exercise is beneficial for the brain, as it can promote the growth of brain nerves and make existing neurons more densely connected. This dense neural network can enable the brain to operate more quickly and efficiently. Furthermore, exercise has been found to improve the symptoms of certain illnesses.^142^, ^143^, ^144^ Research has demonstrated that exosomes derived from skeletal muscle have both paracrine and endocrine effects, which are essential for maintaining muscle equilibrium and intercellular communication.^145^ Exosomes are believed to be essential for allowing muscle‐tissue interaction during exercise.^146^, ^147^ It is widely accepted that the BBB is a significant and impassable barrier that impedes many signaling molecules from the periphery from interacting with the brain. Molecules in the plasma cannot cross the BBB; however, exosomes can cross the BBB through the transcellular route.^148^ It has been suggested that exosomes may contain myokines secreted by skeletal muscle cells during physical activity.^149^ After an hour of cycling, humans have been seen to have higher plasma levels of myokines and proteins found in abundance in small exosomes.^144^, ^150^ Additionally, up to 75% of reported myokines have been located in exosomes,^149^ implying that exosomes may be mediating the release of myokines caused by exercise.

Exosomes, which are filled with a large number of peptides, are secreted from the skeletal muscles of mice.^151^ A pilot study revealed that ALG‐2‐interacting protein X, an exosome marker, is significantly reduced right after acute endurance exercise, indicating the presence of exosomes in muscles and their release in response to exercise. Additionally, a study elucidated the role of myotube‐derived exosomes generated due to muscle atrophy in neuronal dysfunction.^152^ Exosomes containing miR‐29b‐3p that are isolated from long‐term differentiated atrophic C2C12 cells are found to be taken up by neuronal SH‐SY5Y cells, resulting in an increase of miR‐29b‐3p in the recipient cells. The overexpression of miR‐29b‐3p leads to the downregulation of neuronal‐related genes and the inhibition of neuronal differentiation. Additionally, HIF1α‐AS2, a novel c‐FOS targeting lncRNA, is induced by miR‐29b‐3p through the suppression of c‐FOS and is found to be essential for the miR‐29b‐3p‐mediated inhibition of neuronal differentiation. These findings indicate that the circulating miR‐29b‐3p associated with atrophy may mediate the communication between muscle cells and neurons at a distant location.

From Muscle to brain communication, a new field of study could provide substantial new insights and breakthroughs into how metabolism and movement influence the brain condition. Exosomes, as one of the factors (including nucleic acids, peptides, and metabolites) in muscle‐to‐brain communication, can be released into the circulation in response to endurance exercise. All these results above suggest that exosomes may substantially influence the crosstalk between muscle and the brain. Containing microRNA, mRNA, mitochondrial DNA, and peptides, nucleic acids, exosomes might mediate a direct and indirect effect on brain function, which has shown great promise for developing novel therapeutic methods for neurodegenerative diseases and cognitive enhancers for people of all ages. Furthermore, as an exercise signal, exosomes might also monitor the amount and intensity of exercise that is required to prescribe exercise as a booster of neurological and mental health.^146^, ^153^

#### 4.1.3 Lung

Interaction between the brain and lungs is enabled through a variety of pathways, thus forming the brain–lung axis. This bidirectional dialogue between the CNS and the lung could be facilitated by neuroanatomical, endocrine, immune, metabolites and microorganism, and gas pathways.^154^ Studies have demonstrated that exosomes are capable of transmitting messages between the CNS and the nerve cells of the periphery via the cerebrospinal fluid, thus, forming a link between the peripheral circulation and the CNS via the BBB, implying that exosomes and their contents may be employed to facilitate communication between the brain—lung axis. Research has indicated that following TBI, exosomes are present in the circulation in higher amounts than normal, which is linked to acute lung injury (ALI).^155^, ^156^ The uptake of exosomes by pulmonary endothelial cells, which contains proinflammatory cytokines, triggers the inflammasome to release IL‐1β and IL‐18, leading to the apoptosis of these cells.^157^ Exosomes present in the bloodstream of individuals with ischemic stroke may be indicative of a proinflammatory state and could activate macrophages, thus, implying a potential link between exosomes and ALI.^158^

It has been suggested that exosomes originating in the lungs can traverse the BBB to share information with the CNS. Research conducted by Dong‐Xue Gan and his team demonstrated that exosomes secreted from lung cancer cells were taken up by brain vascular endothelial cells, which subsequently caused a decrease in M1 phenotypic microglia and an increase in M2 phenotypic microglia, thus, facilitating the brain metastasis of lung cancer cells.^159^ Recent research has also suggested that exosomes from SARS‐CoV‐2‐infected lungs may transport to areas of the brain associated with neurodegenerative diseases.^160^ Upon arrival, the transcription factors contained in the exosomes influence the neuronal gene regulatory network, potentially accelerating neurodegeneration. These results suggested that exosomes may be communicators between the brain and lung, though the existence of the brain—lung axis is still not generally accepted. Exploring the role of exosomes in bidirectional communication between the lung and the brain not only helps us to understand the brain–lung axis involving lung diseases and CNS diseases but also provides an important target for treatment strategies.^161^ Table 1 reviews the studies reporting the critical roles of exosomes deriving from tissues like the lung, gut and skeletal muscle in communication between the periphery and CNS.

**TABLE 1:** Studies reporting the critical roles of all tissue‐derived exosomes in communication between the periphery and the CNS.

Exosome source	Substance carried	Recipient cell	Disease	Model	Outcome	References
Gut	Virulence factors	Glia	AD	Not given	↑Complement activation ↑Emergence of hallmark proteins ↑Intracerebral inflammation ↑Cognitive decline	^135^
Gut	MicroRNAs	Microglia	I/R	Intestinal I/R in mice	↑Microglial activation ↑Neuronal loss ↑Synaptic stability decline ↑Cognitive impairment	^162^
Muscles	Lipids	Pancreas/Liver	IR	HP‐fed mice	↑Muscle homeostasis	^151^
Muscles	MiR‐29b‐3p	Neuronal cells	Neuronal dysfunction	In vitro	↓Neuronal differentiation ↑Distal communication between muscle and neuronal cells	^152^
Lung cancer cells	Not given	Microglia	Lung cancer metastasis	In vitro	↑M2 phenotypic microglia ↓M1 phenotypic microglia	^159^
Lung	TFs	Neurons	PD/AD	Not given	↓Neuronal gene regulatory network	^160^

Abbreviations. AD, Alzheimer's disease; HP, standard chow diet enriched with 20% palm oil; I/R, ischemia/reperfusion; IR, insulin resistance; PD, Parkinson's disease.; TFs, transcription factors.

### 4.2 Various types of stem cell‐derived exosomes

#### 4.2.1 Mesenchymal stem cells

Mesenchymal stem cells (MSCs) can be released from a variety of sources, including adipose tissue, bone marrow, and the brain. Exosomes, which are derived from MSCs, are a kind of membrane‐bound vesicles that can deliver lipids, proteins, and nucleic acid to other cells and have been found to have a considerable effect on the phenotypic and behavioral characteristics of the target cells, thus, giving a novel insight into cell–cell interaction and communication.^16^, ^163^, ^164^ Recent studies have demonstrated that exosomes isolated from MSC cultures can reproduce the functional effects of MSC treatment.^165^, ^166^ Comparatively, exosomes have comparable effects to those of MSCs when administered.

Exosomes derived from MSCs possess specific traits that differentiate them from other cells, including membrane‐binding proteins, such as CD29, CD44, and CD73, allowing them to be taken up by target cells.^167^ It has been found that exosomal RNA plays a critical role in the regeneration of neurites and the maintenance of neurons in the CNS.^168^ Studies have revealed that the lipids derived from MSC exosomes can facilitate intercellular communication, act as a chemoattractant, and prompt the maturation of dendritic cells.^169^, ^170^

##### Bone marrow‐derived mesenchymal stem cells (BMSCs)

TBI is a primary contributor to mortality and disability in people aged 45 and younger^171^ and is associated with long‐term neurological conditions such as AD and PD. People who have lived through TBI are afflicted with severe neurological and behavioral difficulties and enormous financial and mental repercussions.^172^, ^173^, ^174^ Numerous studies have yielded invaluable information highlighting the advantages of MSC‐derived exosomes in managing TBI.^166^, ^175^, ^176^ Results from Zhang et al. suggest that exosomes enriched with the miR‐17‐92 cluster from human bone marrow MSCs could reduce neuroinflammation, protect hippocampal neurons, and improve sensorimotor and cognitive capacities.^175^, ^177^ Studies have indicated that exosomes from bone marrow MSCs could potentially ameliorate early brain injury (EBI) after subarachnoid hemorrhage, as miRNA129‐5p has anti‐inflammatory and antiapoptotic effects, which can inhibit the HMGB1‐TLR4 pathway.^178^ Despite the lack of comprehension of the exact mechanisms that cause these effects, evidence has indicated that their anti‐inflammatory, angiogenic, neuroprotective, and neurogenic properties may be the primary contributors that account for the observed results in both in vitro and in vivo studies.^179^

Bone MSCs‐derived exosomes are identified to have neuroprotective properties, stimulate angioneurogenesis, and assist with post‐ischemic neurological injury recovery in a mouse stroke experiment.^180^, ^181^, ^182^ Studies conducted on bone MSC exosomes uncovered the presence of miR‐132‐3p and miR‐133b, which may mitigate the effects of ROS production, apoptosis, and tight junction disruption in cells exposed to hypoxia/reoxygenation injury. It has been observed that these effects are connected to neurite remodeling, leading to recovery from a stroke when delivered to astrocytes and neurons, thereby reducing ROS production in cerebral vascular, BBB dysfunction, and brain injury.^183^, ^184^ Research has demonstrated that exosomes are carriers of the miR‐17‐92 cluster, which has a role in neurogenesis, neural remodeling, and oligodendrogenesis in the ischemic boundary region.^175^, ^177^ Hypoxia‐induced exosomes derived from BM‐MSCs are demonstrated to help restore synaptic impairment and reduce inflammation in an APP/PS1 mouse model of AD.^185^

##### Adipose‐derived mesenchymal stem cells (ADMSCs)

Exosomes present in circulation mainly originate from adipose tissue, and having healthy adipose tissue is beneficial for preserving the typical functioning of exosome contents.^186^ It has been reported that exosomes derived from adipose‐derived stem cells (ADSCs) can be used to promote angiogenesis, protect neurons, and increase myelin regeneration, in addition to activating oligodendrocyte progenitor cells to safeguard the brain.^186^, ^187^, ^188^ For example, research has revealed that the systemic inflammation and brain damage caused by sepsis syndrome in rats can be effectively reduced by using allogenic adipose‐derived MSCs‐derived exosomes.^188^ Studies have demonstrated that exosomes derived from ADSCs can reduce the accumulation of β‐amyloid peptides in patients with AD.^189^, ^190^ Research on AD demonstrated that ADSCs secrete exosomes with a large amount of neprilysin, an enzyme used to degrade β‐amyloid peptides in the brain tissue. After transferring these exosomes into neuroblastoma cells, the secreted and intracellular β‐amyloid peptide levels are reduced.^191^ Results of studies have indicated that exosomes extracted from ADSCs, when administered via caudal veins, can reduce the size of infarcts and improve the motor function of ischemic stroke rats.^192^ Research has revealed that MiR‐126‐rich ADSC‐derived exosomes can ameliorate the onset of ischemic stroke, foster neurogenesis, impede microglial activation and ischemic stroke‐induced inflammation, and improve functional recovery.^193^ In addition, MiR‐30d‐5p‐carrying ADSC‐derived exosomes protect acute ischemic stroke by inhibiting inflammatory response and microglial cell polarization mediated by autophagy, thus, reducing the brain injury areas.^194^ Reports suggest that primary neurons exposed to oxygen‐glucose deprivation and co‐cultured with ADSC‐derived exosomes exhibit a marked decrease in neuronal apoptosis, which is thought to be connected to the miR‐25‐3p and miR‐181b‐5p found in exosomes.^195^, ^196^ Research has shown that ADSC‐exosome therapy can be advantageous in safeguarding motor neurons from H_2_O_2_‐induced damage and mortality. This is due to miRNAs such as miR‐21, miR‐222, and miR‐let7a, which can suppress apoptotic pathways, stimulate cell cycle progression, and stimulate proliferation.^197^

Exosomes derived from ADSCs can enhance the functional recovery of TBI when injected intraventricularly into TBI rat models. It has been observed that these exosomes can suppress the production of acute proinflammatory cytokines and activate chronic microglia and macrophages, thus, aiding in the recovery of sensorimotor function in rats.^176^ This mechanism is closely related to exosomal RNA, which can be transported into the brain.

##### Human umbilical cord mesenchymal stem cells (huMSCs)

Different sources of MSCs have been identified, and the human umbilical cord is one of them.^198^ This source has been extensively studied by researchers due to the noninvasive isolation method, the absence of ethical concerns, and the low immunogenicity.^199^ Recent studies have demonstrated that human umbilical cord mesenchymal stem cells (huMSCs)‐derived exosomes have significant impacts on the progression of CNS disorders.^200^, ^201^, ^202^, ^203^, ^204^ An examination by Ding et al.^200^ was conducted to evaluate the effects of huMSCs‐derived exosomes on AβPP/PS1 transgenic mouse models of AD and BV2 cells. The aggregation and formation of Aβ plaque have been observed to stimulate microglia, which is a significant component of neuroimmunoregulation.^205^ In this research, it was observed that the injection of huMSCs‐derived exosomes led to a decrease in Aβ accumulation and an increase in spatial learning and memory in an AD mouse model. In vivo investigations demonstrated that exosomes derived from huMSCs would be able to traverse the BBB and reach the substantia nigra, causing a decrease in the asymmetric rotation induced by apomorphine, a reduction in the number of dopaminergic neurons lost, a decrease in the degree of apoptosis occurring in the substantia nigra, as well as an increase in dopamine levels in the striatum.^201^ The results suggest that huMSCs‐derived exosomes may be able to traverse the BBB and, therefore, have a treatment capability for PD, demonstrating their potential for a successful treatment.

Recent evidence has pointed to the potential therapeutic effects of exosomes derived from MSCs in preclinical rodent models of stroke and brain injury. Studies have established that exosomes secreted from huMSCs in standard culture conditions can ameliorate the damage caused by ischemia.^204^ Exosomes from huMSCs have been identified as a potential therapeutic approach for subarachnoid hemorrhage (SAH)‐induced EBI, hypoxic‐ischemic brain damage (HIBD)^206^ and TBI,^203^ as they have been found to prevent EBI by suppressing apoptosis through the BDNF/TrkB/CREB signaling pathway,^207^ the p38 MAPK/STAT3 signaling pathway^208^ and FOXO3a‐dependent mitophagy^206^ regulated by miRNAs.

Spinal cord injury (SCI) is a severe traumatic CNS disorder that often leads to sensory and motor deficits in the area below the injury. Numerous studies have indicated that stem cell‐based therapy is a viable approach to treating SCI^209^ ^.^Results of the study suggested that exosomes derived from huMSCs, modified by miR‐146a‐5p, demonstrated a greater capacity to enhance neurological function in rats with SCI than their unmodified counterparts due to their ability to specifically target neurotoxic astrocytes.^202^ Similarly, huMSCs‐derived exosomes, transferring miR‐199a‐3p/145‐5p into neurons of SCI rats, were found to influence TrkA ubiquitination and consequently activate the NGF/TrkA signaling pathway.^210^ This suggests that exosomes may be an effective therapeutic approach for SCI. In addition to astrocytes and neurons, exosomes may have a beneficial effect on motor function by modulating the BCL2/Bax and Wnt/β‐catenin signaling pathways in microglia, thereby providing anti‐apoptotic and anti‐inflammatory benefits.^211^ The research findings indicate that exosomes derived from huMSCs may serve as a potential bridge of communication between the periphery and CNS.

#### 4.2.2 Other types of stem cells

Human dental pulp mesenchymal stem or stromal cells (DP‐MSCs) and their exosomes, due to their neurotropic character, have been proposed to be a promising therapeutic approach for the alleviation of symptoms of neurodegenerative diseases and other hard‐to‐treat maladies. To evaluate the efficacy of human DP‐MSCs and human BMSCs, Song et al. conducted a research study in a stroke model of rats.^212^ Compared with control rats, intravenous injection of both DP‐MSCs and BMSCs into rats after middle cerebral artery occlusion can reduce infarct volume and improve functional recovery, and the DP‐MSCs showed a better therapeutic effect.

In addition to DP‐MSCs, mesenchymal stem or stromal cells can be obtained from a single tooth via various dental tissue‐derived stem cells, including periodontal ligament stem cells, stem cells from human exfoliated deciduous teeth (SHED), stem cells from apical papilla, and dental follicle progenitor cells.^213^ Deciduous teeth are advantageous for regenerative medicine, as they share similar embryonic characteristics compared to stem cells from adult teeth. Recent research has suggested that exosomes derived from SHED may be a promising form of therapy for neurological disorders. Li et al. demonstrated that SHED‐derived exosomes could contribute to functional recovery after TBI by altering the microglia M1/M2 polarization in rats.^214^ Following TBI, proinflammatory effects are triggered by the secretion of inflammatory factors, which can lead to a chronic state if the proinflammatory process is not adequately inhibited. Therefore, shifting microglia from M1 to M2 is seen as a potential target for TBI treatment.^215^ Similar to neural stem cells, SHED exosomes have been found to modulate the host microglia in a manner that supports tissue repair and suppresses pro‐inflammatory mediators.

The potential of NPCs to treat neurological disorders has been demonstrated through the secretion of EVs. However, the limited number of NPCs in the brain restricts further utilization of EVs for treatment. To overcome this limitation, a somatic reprogramming approach has been employed to generate iNPCs from somatic cells such as mouse fibroblasts and astrocytes. Subsequent studies suggest that the exosomes (iEXOs) derived from iNPCs have a distinct capacity to promote the proliferation of NPCs in comparison to exosomes. This raises the hypothesis that exosomes and iEXOs may be able to exert their therapeutic effects in neurogenesis through the transfer of critical miRNAs, thus, providing a promising avenue for the development of effective cell‐free therapeutic strategies for neurological disorders.^216^ A subsequent study demonstrated that EVs derived from iNPCs could enhance the proliferative capacity of wild‐type NPCs. Proteomics analysis of the EV contents revealed that iNPC‐EVs contained higher concentrations of proteins associated with growth factors, which were thought to activate the extracellular signal‐regulated kinase (ERK) pathways.^217^ Taken together, these findings suggest that iNPC‐derived EVs may be a viable therapeutic approach for neurological diseases, as they are capable of stimulating NPC proliferation, releasing growth factors, and activating ERK pathways. Table 2 reviews the studies reporting the critical roles of exosomes deriving from various stem cell types in communication between the periphery and CNS.

**TABLE 2:** Studies reporting the critical roles of various types of stem cell‐derived exosomes in communication between the periphery and the CNS.

Exosome source	Substance carried	Recipient cell	Disease	Model	Outcome	Ref.
BMSCs	Not given	Neurons/Microglia	TBI	TBI in rats	↑Spatial learning ↑Neurogenesis in the dentate gyrus ↓Neurological deficits ↓Foot‐fault frequency ↓Brain inflammation	^166^
BMSCs	MiR‐17‐92	Neurons	TBI	CCI‐TBI in rats	↑Sensorimotor and cognitive function ↑Angiogenesis and neurogenesis ↓Neuroinflammation ↓Hippocampal neuronal cell loss	^175^
BMSCs	MiR‐17‐92	Neurons	Stroke	MCAO in rats	↑Neurite remodeling ↑Neuronal dendritic plasticity ↑Neurogenesis and oligodendrogenesis ↑PI3K/Akt/mTOR signaling pathway ↓PTEN level	^177^
BMSCs	RNAs/Proteins	Neurons/Microglia	EBI after SAH	SAH in rats	↑Neurological functions ↑BBB integrity ↓Brain water content ↓HMGB1‐TLR4 signaling pathway ↓Inflammation ↓Apoptosis	^178^
BMSCs	MiR‐124	Cortical neural progenitors	IS	Photothrombosis in mice	↑Neurogenesis	^182^
BMSCs	Not given	Astrocytes/ Neurons	Stroke	MCAO in rats	↑Functional recovery ↑Axonal plasticity ↑Neurite remodeling	^183^
BMSCs	MiR‐132‐3p	BMECs	IS	MCAO in mice	↑ZO‐1 and Claudin‐5 expression ↑PI3K/Akt/eNOS signaling pathway ↓Cerebral injury ↓Permeability ↓ROS production and apoptosis ↓BBB disruption	^184^
BMSCs	Not given	Astrocytes/ Microglia	AD	APP/PS1 mice	↑Learning and memory capabilities ↑Restoration of synaptic dysfunction ↓Inflammatory responses	^185^
ADSCs	Not given	Microglia	Neural injury	In vitro	↓Microglial activation ↓NF‐kB and MAPK signaling pathway ↓Cytotoxicity of activated microglia ↓Neural injury	^188^
hADSCs	Neprilysin	N2a cells	AD	In vitro	↓Secreted Aβ40 and Aβ42 levels ↓Intracellular Aβ42 level	^189^, ^191^
ADMSCs	Not given	Oligodendrocyte	Stroke	Subcortical infarct model in rats	↑Functional recovery ↑Fiber tract integrity ↑Axonal sprouting ↑White matter repair markers ↑ White matter integrity	^192^
ADSCs	MiR‐126	Neurons/ Microglia	Stroke	MCAO in rats	↑Functional recovery ↑Neurogenesis ↓Neuroinflammation	^193^
ADSCs	MiR‐30d‐5p	Microglia	AIS	AIS in rats	↑M2 microglia/macrophage polarization ↓Cerebral injury area of infarction ↓Autophagy	^194^
ADSCs	MiR‐181b‐5p	BMECs	Stroke	MCAO in rats	↑Angiogenesis of BMECs	^195^
ADMSCs	MiR‐25‐3p	Neurons	Stroke	MCAO in mice	↑Neuroprotection ↑Autophagic flux	^196^
ADSCs	Not given	Neurons	ALS	ALS model in vitro	↓Mitochondrial dysfunction ↓SOD‐1 aggregation	^197^
hADSCs	Not given	Microglia/ Macrophages	TBI	TBI in rats	↑Functional recovery ↑Neurogenesis ↓Neuroinflammation ↓Neuronal apoptosis	^176^
huMSCs	Not given	Microglia	Perinatal brain injury	Perinatal brain injury in rats	↓TLR4/CD14 signaling pathway ↓Proinflammatory molecules production ↓Microgliosis	^218^
huMSCs	Not given	Microglia	HIBD	In vitro	↑FOXO3a‐dependent mitophagy ↓Microglial pyroptosis	^206^
huMSCs	miR‐26b‐5p	PC12 cells	EBI after SAH	SAH in rats	↓p38 MAPK/STAT3 signaling pathway ↓OxyHb‐induced cell injury	^208^
huMSCs	miR‐206 modified	Neurons	EBI after SAH	SAH in rats	↑Neurological deficit ↑BDNF/TrkB/CREB signaling pathway ↓Brain edema ↓Neuronal apoptosis	^207^
huMSCs	Not given	Microglia	AD	AβPP/PS1 mice	↓Cognitive dysfunctions ↓Aβ deposition ↓Neuroinflammation.	^200^
huMSCs	Not given	Neurons	PD	PD rats	↑Proliferation of SH‐SY5Y cells ↑Autophagy ↑Dopamine level in the striatum ↓Apomorphine‐induced asymmetric rotation ↓Neuron loss and apoptosis	^201^
huMSCs	miR‐146a‐5p	Astrocytes	SCI	SCI in rats	↑Neural tissue preservation ↑Locomotor function of the hindlimbs ↓Inflammatory responses ↓Neurotoxic astrocytes numbers ↓Traf6/Irak1/NF‐κB signaling pathway	^202^
huMSCs	miR‐199a‐3p/145‐5p	Neurons	SCI	SCI in rats	↑NGF/TrkA signaling pathway ↑Locomotor function of SCI rats	^210^
huMSCs	Not given	Neurons/ Microglia	SCI	SCI in rats	↑Motor function of SCI rats ↑Wnt/β‐Catenin signaling pathway ↓Apoptosis ↓Inflammatory state from SCI injury	^211^
huMSCs	Not given	Astrocytes/ Microglia	TBI	TBI in rats	↑Neurological function recovery ↓Neuronal apoptosis ↓Microglia and astrocytes activation	^203^
huMSCs	Not given	Neurons	Stroke	MCAO in rats	↓Infarct size ↓Ischemic brain damage	^204^
DP‐MSCs	Not given	Neurons/ Astrocytes	Stroke	MCAO in rats	↑Functional recovery ↓Infarct volume ↓Reactive gliosis ↓MAPK and TGF‐β signaling pathway	^212^, ^219^
SHED	Not given	Microglia	TBI	TBI rats via the “free‐falling method”	↑Motor functional recovery ↑M2 phenotypic microglia ↓Neuroinflammation ↓M1 phenotypic microglia	^220^, ^221^
iNPCs	miR‐21a	Neurons	Not given	In vitro	↑Neurogenesis	^216^
iNPCs	Not given	Not given	Not given	Not given	↑ERK signaling pathway ↑Growth factor‐associated proteins levels	^217^

Abbreviations. AD, Alzheimer's disease; ADMSCs, adipose‐derived mesenchymal stem cells; ADSCs, adipose‐derived stem cells; AIS, acute ischemic stroke; ALS, amyotrophic lateral sclerosis; BMECs, brain microvascular endothelial cells; BMSCs, bone marrow‐derived mesenchymal stem cells; CCI, controlled cortical impact; DP‐MSCs, dental pulp mesenchymal stem/stromal cells; EBI, early brain injury; hADSCs, human adipose tissue‐derived MSCs; HIBD, hypoxic‐ischemic brain damage; huMSCs, human umbilical cord mesenchymal stem cells; iNPCs, induced neural stem/progenitor cells.; IS, ischemic stroke; MCAO, middle cerebral artery occlusion; PD, Parkinson's disease; SAH, subarachnoid hemorrhage; SCI, spinal cord injury; SHED, stem cells from human exfoliated deciduous teeth; TBI, traumatic brain injury.

## 5 EXOSOMES AS THERAPEUTICS VEHICLES FOR BRAIN

Exosomes feature low toxicity, biodegradability, and the ability to encapsulate drugs and endogenous biologically active molecules and cross the BBB.^222^ What's more, compared with other synthetic nanoparticles, the major advantage of exosomes is their nonimmunogenic nature, leading to a long and stable circulation, show great promise as therapeutic vehicles and biological indicators for brain diseases. The utilization of exosomes as a drug delivery system (DDS) to the brain has been strengthened in recent years, as they are capable of crossing the BBB.^223^, ^224^, ^225^ Several studies have demonstrated remarkable preclinical success in addressing unmet clinical needs. In particular, a study used exosomes loaded with rhodamine and anticancer drugs to show their ability to cross the BBB, and the transport mechanisms were then examined using zebra fish as an animal model. To assess the capability of exosomes to transfer drugs across the BBB in vivo, bEND.3‐derived exosomes loaded with rhodamine and doxorubicin, or paclitaxel, are injected into zebra fish embryos. After the experiment, brain tissue is inspected for rhodamine fluorescence, implying the potential of exosomes to deliver drugs. Subsequently, a primary brain cancer model is established in zebra fish, and the efficacy of drugs with and without exosomes is compared. Results from the study indicated that the zebra fish brain model treated with exosomes loaded with doxorubicin demonstrated a significant therapeutic efficacy compared to doxorubicin alone. Additionally, research on AD treatments has revealed that the administration of curcumin‐loaded exosomes has yielded more significant neuroprotective effects than free curcumin.^226^ Qi et al. investigated the effects of free quercetin and quercetin‐loaded exosomes on AD models and discovered that the exosome delivery system is more beneficial than free quercetin in terms of permeability across brain parenchymal tissues and cognitive function improvement.^227^ Recent studies have indicated that exosomes loaded with the antioxidant protein catalase can be effectively delivered across the BBB, leading to a beneficial outcome in PD. Intranasal administration of catalase‐loaded exosomes effectively shields dopamine neurons in the substantia nigra pars compacta from oxidative stress in the PD mouse brain. In contrast, intravenous treatment with dopamine‐loaded serum‐derived exosomes significantly impacted PD mouse models.^228^ Additionally, research has suggested that neurotoxicity and neuroinflammation in PD models could be improved by implanting mRNA‐loaded engineered exosomes.^229^ Moreover, multiple clinical trials have been carried out to evaluate the effectiveness of exosomes as endogenous drug delivery vesicles, and the outcomes have been encouraging.^223^, ^230^

It is noted that natural exosomes show poor targeting ability, which greatly reduces the therapeutic effect. Engineering technology opens the possibility of obtaining exosomes with the active targeting ability to accumulate in specific cell types and tissues by membrane modification or loading functional groups into cavities. By engineering exosomes to present surface ligands, receptor‐mediated tissue targeting can be achieved, and signaling events in recipient cells can be induced or inhibited. It has been proven that the expression of BACE1 in the brain can be inhibited by intravenously administering exosomes derived from RVG‐modified dendritic cells, which contain therapeutic Bace1‐targeting siRNA, to mice. This is a potential therapeutic approach for AD. Investigations have revealed that exosomes modified with RVG and containing siRNA targeting α‐synuclein were successful in curtailing aggregate formation in the brains of S129D a‐synuclein mice and ameliorating brain pathology. Moreover, the c(RGDyK) peptide has been combined with the exosome to be employed for cerebral ischemia therapy. Following the intravenous administration of c(RGDyK)‐conjugated exosomes (cRGD‐Exo) containing curcumin, a notable decrease in the inflammatory response and cellular apoptosis was observed in the area of the ischemic brain that had been effectively targeted.^231^ Another example is the engineered exosomes constructed by incubating iron oxide nanoparticles with MSCs to obtain magnetic nanovesicles (MNVs), with potential for in vivo magnetic targeting.^232^ Under the guidance of an external magnetic field, the MNVs can achieve targeting ischemic lesions in the brain. In short, the engineered exosomes with targeting ability can increase the accumulation of exosomes in the location of brain, thus, avoiding damage to other organs, expanding the application of exosomes in brain diseases.

## 6 CHALLENGES AND FUTURE DIRECTIONS IN EXOSOME RESEARCH

Exosomes as an emerging area, are moving rapidly. Meanwhile, the advancement of other technologies, like engineering technology, imaging technology, along with the analytical technologies will greatly boost the development of exosomes. As described above, engineered exosomes are a promising tool delivering messages or drugs to specific cells to realize precise therapy. Currently, researchers have developed multiple strategies to engineer natural exosomes to achieve more functions including the technologies of genetic engineering, biochemical engineering, and physical engineering. All these engineering technologies will improve the targeting functions and optimize the tracing techniques, interaction with the BBB, and therapeutic effects, finally opening opportunities of the clinical transformation.^233^

Tracing and imaging technology to monitor exosomes in vivo is another technology to improve the development of exosomes. Currently, there still exist many mysteries surrounding the manner in which the exosomes achieve their intended task. The process of how the exosomes exert therapeutic effects from injection is a black box, and plenty of information remains unknown. Tracing and imaging technology can provide important knowledge about their biodistribution, migration abilities, toxicity, biological role, communication capabilities, and mechanism of action. Therefore, the development of sensitive, efficient, and biocompatible exosome imaging techniques is highly desired to open the “black box.” Much efforts have been made to develop different strategies for exosome labeling and imaging, allowing for monitoring their physiological functions, biodistribution, and targeting mechanisms in vivo. These imaging techniques may greatly advance exosome applications, which mainly include fluorescence imaging, bioluminescence imaging, radionuclide and magnetic resonance imaging techniques. For example, Takahashi Y et al. first reported that exosomes labeled with the bioluminescent reporter protein could achieve visualization in vivo.^234^ Through in vivo imaging, we can see B16‐BL6 exosomes were rapidly discharged from the blood circulation with a half‐life of about 2 min. In addition, signals from the B16‐BL6 exosome first appeared in the liver and then in the lung. After that, the labeled exosomes with a fusion protein of Gaussian luciferase and gLuc‐lacherherin were reported by Takahashi Y et al. They also successfully evaluated the tissue distribution of exosomes in mice by bioluminescence detection.^235^ It is worth mentioning that the second near‐infrared (NIR‐II) fluorescence imaging technique. Imaging in the “near‐infrared window” (1000–1700 nm) is an emerging imaging technique with the advantages of low light scattering and deeper tissue penetration ability, as well as penetrating the skull to track the exosomes in the brain.

The potential of exosomes to be used as disease biomarkers and therapeutic agents has been widely acknowledged. However, there are still significant challenges to be overcome before exosomes can be used in a clinical setting. Standardization and quality control are two of the most pressing issues, as exosomes are typically isolated using ultracentrifugation‐based methods,^84^ which can result in complex mixtures of EVs and other components of the extracellular space.^28^, ^236^ The combination of different isolation techniques can indeed improve the purity of exosomes, but it also inescapably increases the cost and difficulty of operation and leads to decreases in overall yield and unreliable downstream analysis. Therefore, prior to selecting an isolation strategy, it is necessary to carefully consider the nature of the sample and the purpose of the study to choose an appropriate combination of techniques. Furthermore, the components of exosomes may vary depending on their origin and cell types, making it challenging to ensure that the surface markers and cargo of exosomes are 100% pure and homogeneous. Therefore, there is a need to develop standardized good manufacturing practices for their processing and characterization that can differentiate the different types of EVs and obtain high‐purity exosomes in a rapid, efficient, reproducible, and clinically friendly manner.^27^, ^30^ As natural drug delivery vectors, loading drugs into exosomes, surface modification to structure DDS or engineered exosomes also face challenge. It is necessary to ensure that the engineered exosomes are stable, have enough loading efficiency, and are homogeneous. Moreover, there is a problem of transferring undesirable material that is produced by the cells, which can be immunogenic and carcinogenic.^49^, ^237^

Ultimately, as an emerging research field, the use of exosomes lacks management and standardization, which may lead to unexpected issues. Thus, research into exosomes must take into account the ethical and commercial implications.

## 7 CONCLUSION AND FUTURE PERSPECTIVES

Exosomes are an increasingly investigated topic. Over the last decade, much efforts have been made in understanding the biogenesis, content, and biological function of exosomes. And new functions and mechanisms are continuously revealed. They are capable of transferring their cargo of proteins, lipids, and transcriptional factors to target cells, serving a fundamental role in intercellular communication. Furthermore, Accumulating evidence suggests exosomes have the potential to cross the BBB, thereby allowing communication between the additional organs such as the gut, bone, adipose tissue, skeletal muscle, lung, and CNS. In addition to these advantages, exosomes are biocompatible, low immunogenic, stable, have a long half‐life, and show great promise as therapeutic vehicles for the brain. As a result, they may be utilized therapeutically, and new strategies are being devised to exploit them. To promote the successful transformation of exosomes into clinical applications, many engineering strategies have been developed to improve the targeting ability and treatment effect. With the advancement of engineering and imaging technology, more latent functions and mechanisms will be found, and more complex diseases are anticipated to be diagnosed and treated efficiently.

**FIGURE 4:**
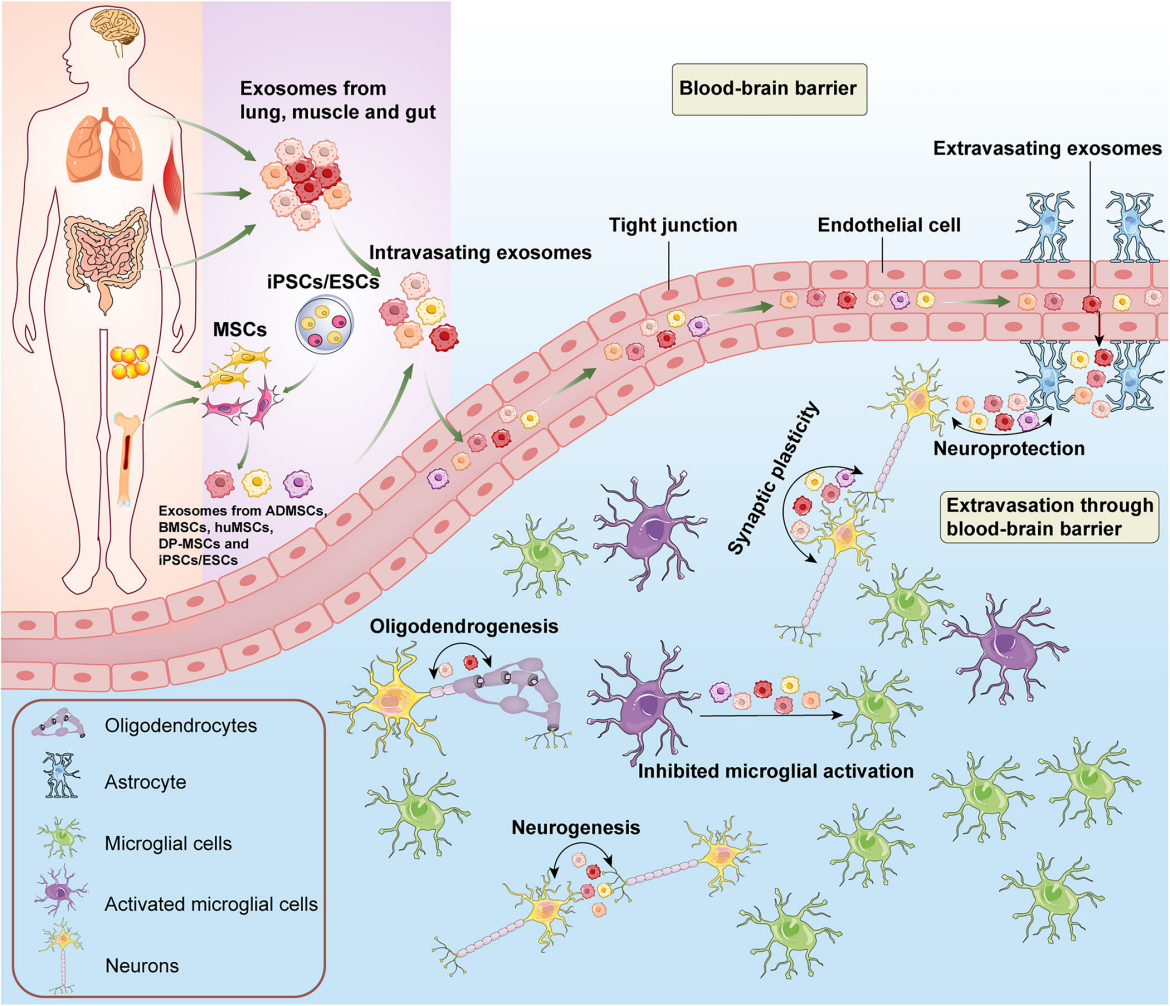
Schematic representation of the critical role of peripheral exosomes in the communication between neural cells. Exosomes, derived from external sources, like the lung, gut, skeletal muscle and various stem cell types, can traverse the blood–brain barrier and be taken up by neurons and glial cells in the central nervous system. Exosomes, as illustrated in Figure 1, possess a range of constituents that can be applied to reduce misfolded proteins, inflammation, tissue injury, and diseases. In cases of injury, including stroke or TBI, exosomes can enhance synaptic activity, neural survival, neurite outgrowth, and neurogenesis while limiting microglial activation, thus, providing neuroprotective benefits. MSCs, mesenchymal stem cells; ADMSCs, adipose‐derived mesenchymal stem cells; BMSCs, bone marrow‐derived mesenchymal stem cells; huMSCs, human umbilical cord mesenchymal stem cells; DP‐MSCs, dental pulp mesenchymal stem/stromal cells; iPSCs, induced pluripotent stem cells; ESCs, embryonic stem cells.

**FIGURE 5:**
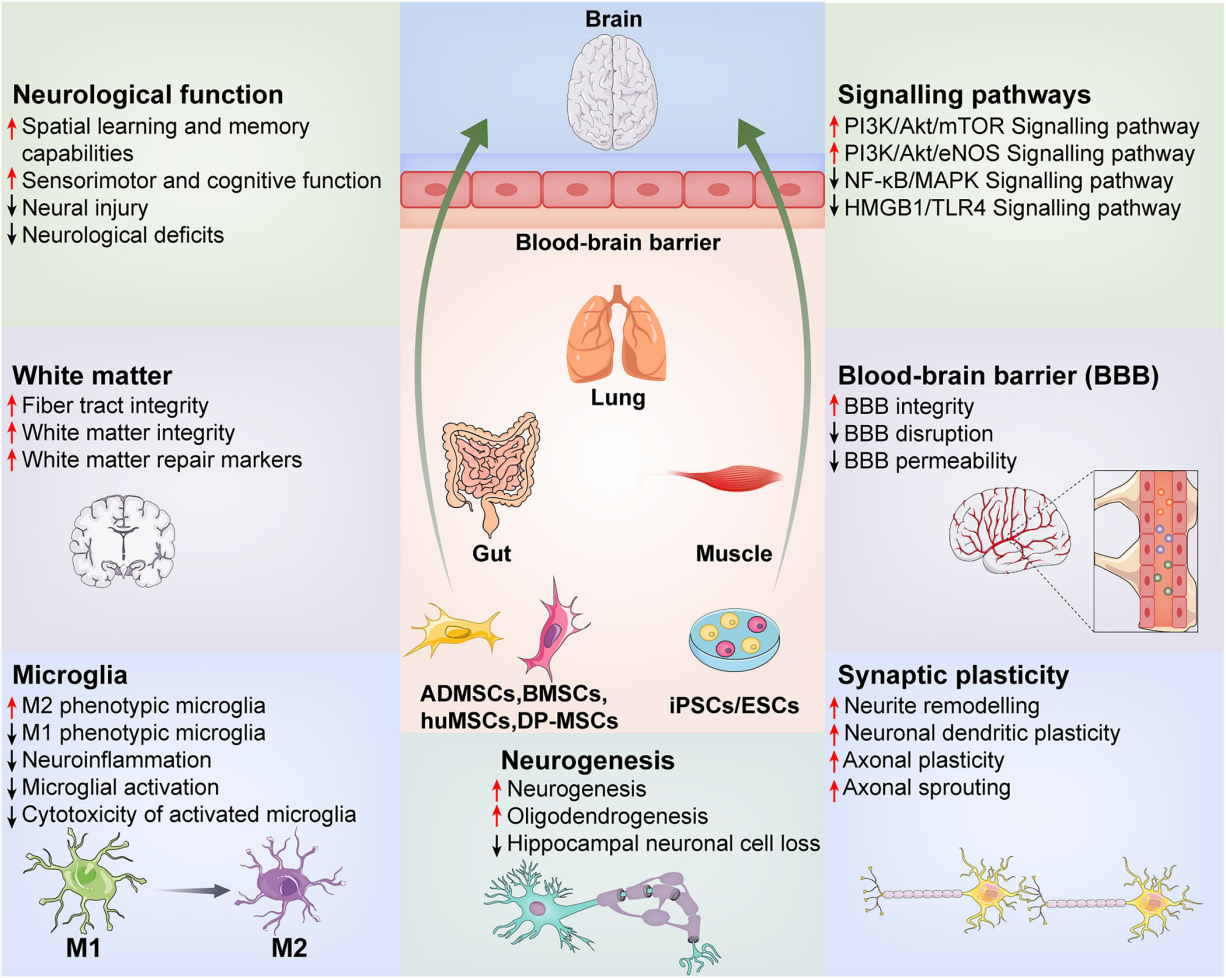
Mechanisms of peripheral exosomes in the communication between neural cells. Exosomes, derived from peripheral sources, such as the lung, gut, skeletal muscle tissue and various stem cell types, possess the ability to cross the blood–brain barrier and enter the central nervous system. For conditions caused by trauma, such as TBI or stroke, research has demonstrated that exosomes can restore neurological function, protect the blood–brain barrier and white matter, encourage synaptic growth, induce neurogenesis and suppress neuroinflammation by modulating different pathways. Consequently, exosomes are crucial in forming a connection between the periphery and the CNS. ADMSCs, adipose‐derived mesenchymal stem cells; BMSCs, bone marrow‐derived mesenchymal stem cells; huMSCs, human umbilical cord mesenchymal stem cells; DP‐MSCs, dental pulp mesenchymal stem/stromal cells; iPSCs, induced pluripotent stem cells; ESCs, embryonic stem cells.

In this review, we focus on exploring the communication between peripheral exosomes derived from various tissues and stem cells and the CNS (Figure  4), as well as the potential mechanism of peripheral exosomes affect the brain's functioning (Figure  5). As the communication between the CNS and periphery is bidirectional, exosomes secreted by neural cells may also cross the BBB and potentially play equally important roles in the periphery. Although the related literature is relatively sparse, recent researches detected the brain‐derived EVs in plasma. And astrocyte‐derived exosomes are capable of transferring miRNA to metastatic tumor cells, suggesting that brain‐derived EVs may transfer molecular information to tissues remote from the CNS.^238^, ^239^, ^240^ In the future, more research is needed to study the role and mechanism of exosomes for bidirectional communication between the CNS and periphery.

Overall, exosomes are a promising field that is advancing at a rapid pace, and answers to pressing questions may soon be discovered. As for the aspects of clinical transformation, they show great potential in the application of diagnosis, treatment, and drug delivery, based on the characteristics and functions of exosomes. In terms of diagnosis, the use of body fluid samples to detect exosomes as biomarkers can obtain disease‐related information such as early diagnosis of tumors and prognostic evaluation of efficacy. As for the treatment of disease, exosomes have also become a research hotspot. More and more enterprises are developing exosome therapeutics and actively carrying out relevant clinical trials for the treatment of tumor, diabetes, and cardiovascular disease. In terms of drug delivery, exosomes have become a new drug delivery method in addition to viral vectors, lipid nanoparticles, and other delivery methods, which are rushed by many drug research and development enterprises. Anti‐inflammatory agent (curcumin) and anticancer agent (adriamycin and paclitaxel) have been loaded into exosome vesicles for the treatment of corresponding diseases. Despite the existing obstacles mentioned above, the future clinical transformation of exosomes is inspiring.

DECLARATIONS

## AUTHOR CONTRIBUTIONS

WXH and HLZ drafted the manuscript and prepared the figures and tables. LF and CMC revised the manuscript. RLD and JW collected the related references. PJ designed the study. All authors read and approved the final manuscript. WXH and HLZ contributed equally to this work.

## CONFLICT OF INTEREST STATEMENT

The authors declare they have no conflict of interest.

## ETHICS APPROVAL

Not applicable.

## Data Availability

All data relevant to this review are included in the text, references, table, and figures.
